# Supplementary material for: Novel Repositioning Therapy for Drug-Resistant Glioblastoma: In Vivo Validation Study of Clindamycin Treatment Targeting the mTOR Pathway and Combination Therapy with Temozolomide
Source: Cancers (Basel). 2022 Feb 2;14(3):770. doi: 10.3390/cancers14030770 (PMC8833675; doi:10.3390/cancers14030770)
Supplement: Supplementary file 1 [file cancers-14-00770-s001.zip › cancers-1544483-supplementary.pdf]

cancers MDPI

Supplementary Materials

Novel Repositioning Therapy for Drug-Resistant Glioblastoma: In Vivo Validation Study of Clindamycin Treatment Targeting the mTOR Pathway and Combination Therapy with Temozolomide

Takeyoshi Eda, Masayasu Okada, Ryosuke Ogura, Yoshihiro Tsukamoto, Yu Kanemaru, Jun Watanabe, Jotaro On, Hiroshi Aoki, Makoto Oishi, Nobuyuki Takei, Yukihiko Fujii and Manabu Natsumeda

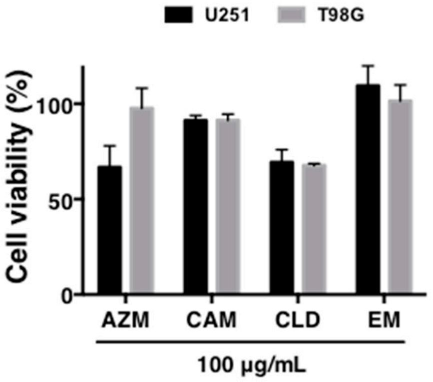

Figure S1: Effect of approved macrolide antibiotics on cell proliferation of U251 and T98G cells.

The cytotoxic effect of approved macrolide antibiotics was measured by WST-1 cell proliferation assay. Cells were treated with four approved antibiotics, azithromycin (AZM), clarithromycin (CAM), clindamycin (CLD), and erythromycin (EM) (each 100 μg/mL) for 72 h and subjected to the WST-1 assay. The viability of untreated cells was considered 100%. Data reveals % ratio to the final number of control cultures as mean ± SE.

A

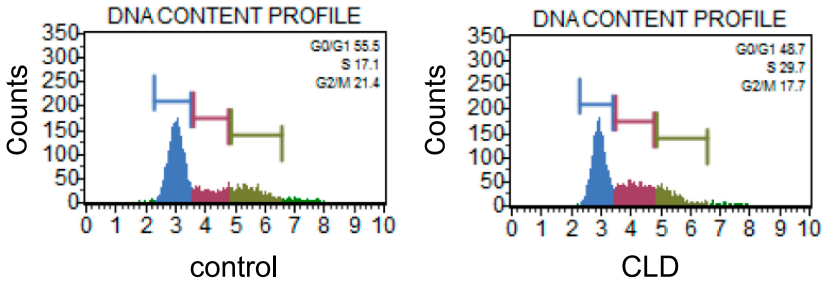

B

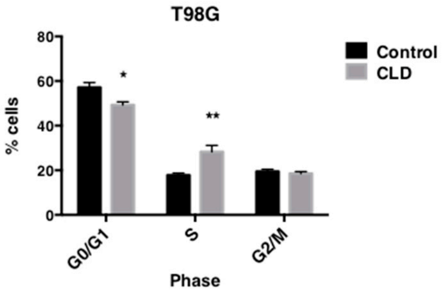

Figure S2 Effect of clindamycin on cell cycle progression in T98G cells

Cell cycle analysis after treatment with CLD in T98G. Cells were treated with CLD (440 μM) for 24 h and subjected to cell cycle analysis. Cells were classified three phases (A). The graph represent % cells in G0/G1, S, and G2/M phase (B), revealed as mean ± SE. Results shown were representative four independent experiments. (* $P < 0.05$, and ** $P < 0.01$ vs. control: *t*-test)

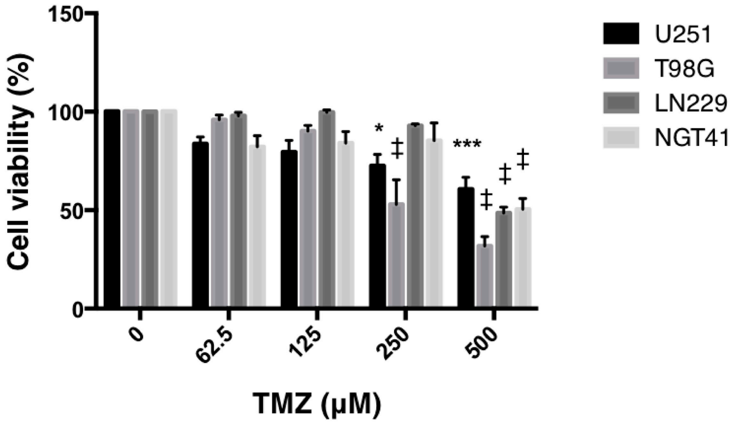

Figure S3 Dose response of temozolomide on cell proliferation in glioblastoma cell lines

The cytotoxic effect of temozolomide (TMZ) was measured by WST-1 assay. Cells were treated with TMZ (0, 62.5, 125, 250, and 500 μM) for 72 h and subjected to the WST-1 cell proliferation assay. The viability of untreated cells was considered 100%. Data are reveals % ratio to the final cell number of control in each cell lines as mean ± SE, similar results shown were representative four independent experiments. (* $P < 0.05$, *** $P < 0.001$, ‡ $P < 0.0001$: two-way ANOVA)

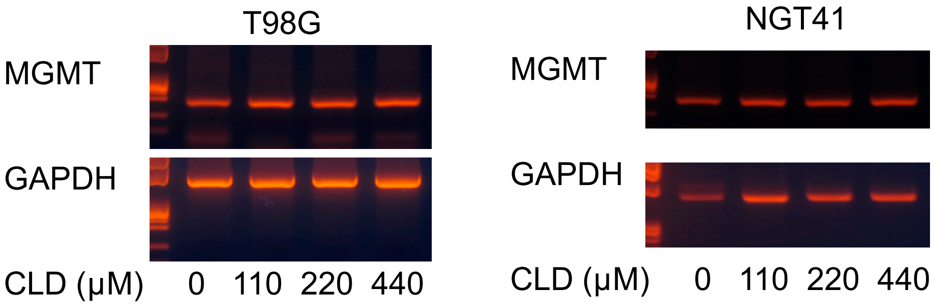

***Figure S4** Analysis of MGMT mRNA expression*

Total RNA was isolated from cell lysate with the guanidinium-phenol solution (ISOGEN; Nippon Gene, Osaka, Japan). Total RNA was converted to cDNA and MGMT was selectively amplified by a RT-PCR kit (One step SYBR PrimeScript PLUS, Takara Bio Inc., Shiga, Japan) using the forward primer (5′-GTGGCCTCACGATGTGTATG) and the reverse primer (5′-ACCCGCTGTGTGACTTATCC). RT-PCR of glyceraldehyde-3-phosphate dehydrogenase (GAPDH) mRNA was similarly carried out with the forward primer (5′-GTCAGTGGTGGACCTGACCT) and the reverse primer (5′-AGGGGTCTACATGGCAACTG). GAPDH was used as an internal control. PCR amplification was performed with the 30 cycles of 95°C for 5 sec and 60°C for 30 sec and products were detected with Gel Red staining (Wako Chemicals Inc., Tokyo, Japan). The amplified products were separated by agarose electrophoresis and visualized by a UV transilluminator.

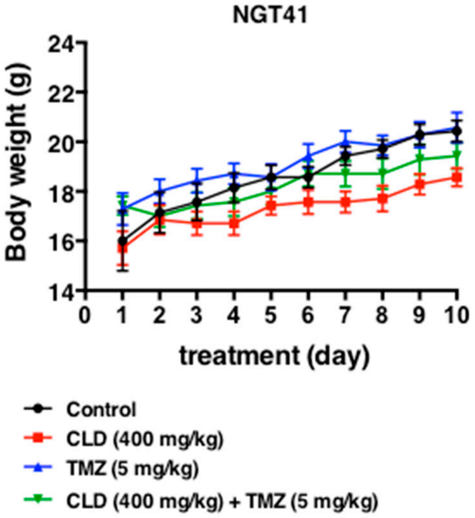

Figure S5 Measurement of mice weight during a treatment period with clindamycin and temozolomide

Mice were weighed daily to determine the dose. In addition, general condition of mice was closely observed to investigate the adverse events caused by the medication. The data is expressed as mean ± SE (N = 7).

Figure S6. Original western bolt.

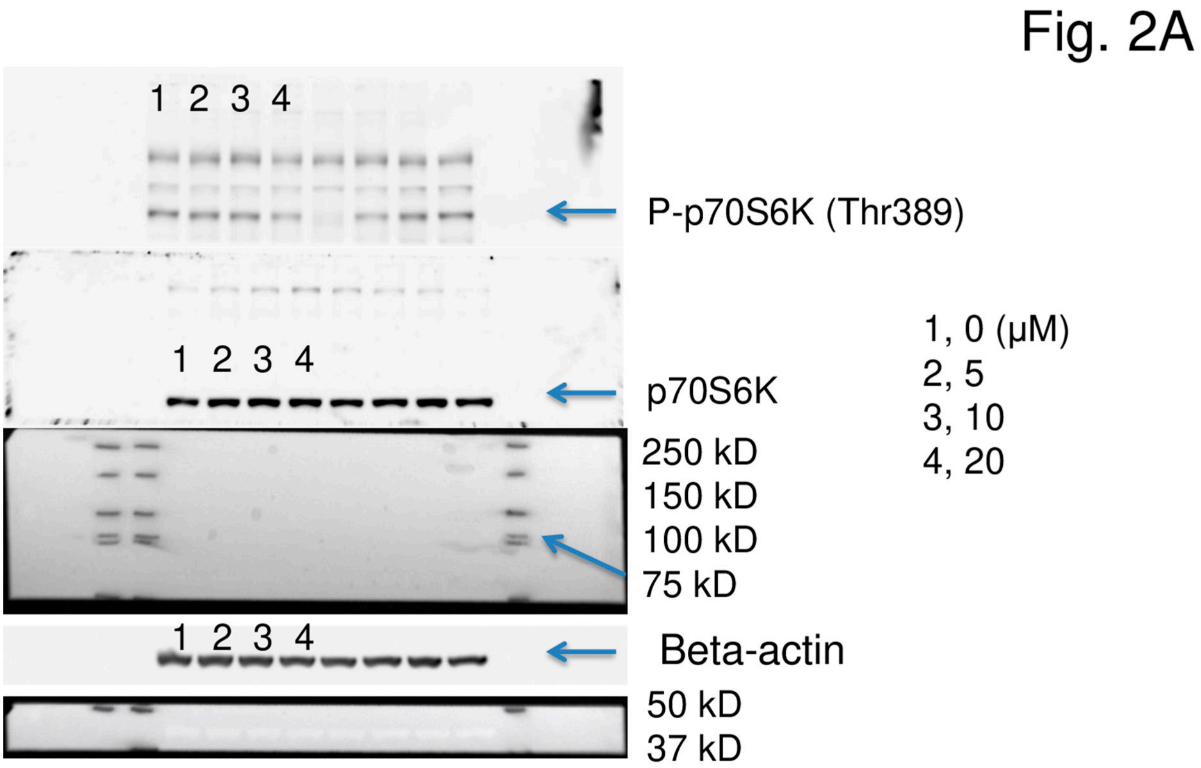

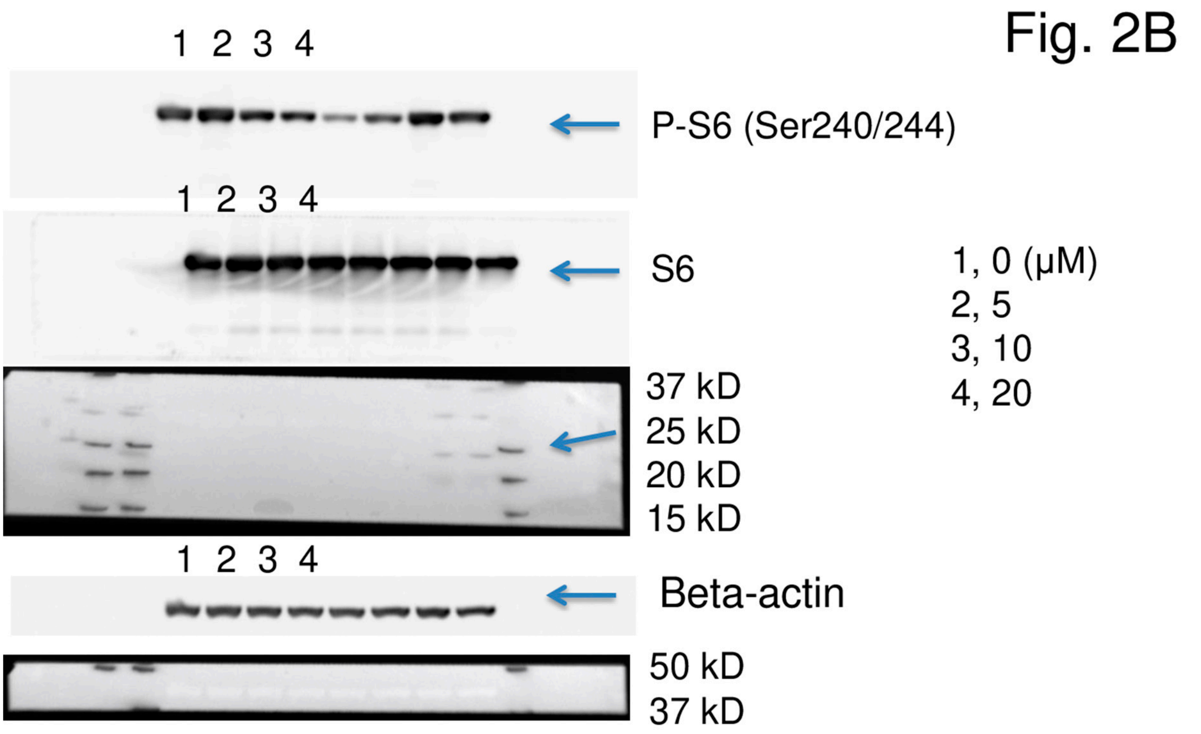

Fig. 2C

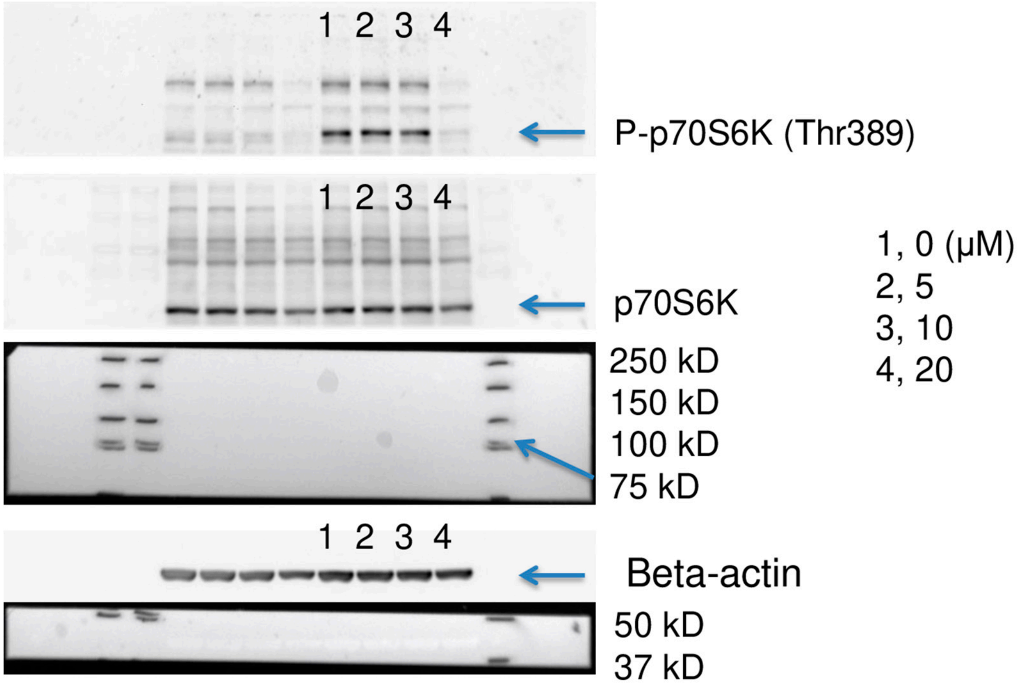
1 2 3 4
P-p70S6K (Thr389)
1 2 3 4
p70S6K
250 kD
150 kD
100 kD
75 kD
1 2 3 4
Beta-actin
50 kD
37 kD
1, 0 (µM)
2, 5
3, 10
4, 20

Fig. 2D

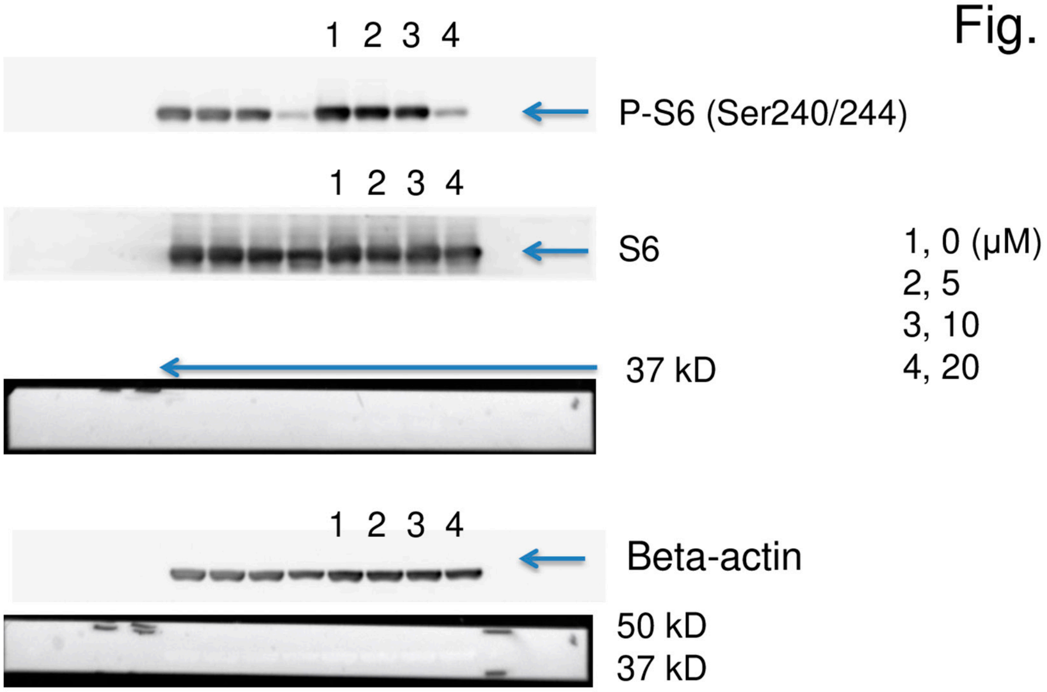
1 2 3 4
P-S6 (Ser240/244)
1 2 3 4
S6
37 kD
1 2 3 4
Beta-actin
50 kD
37 kD
1, 0 (µM)
2, 5
3, 10
4, 20

Fig. 2E

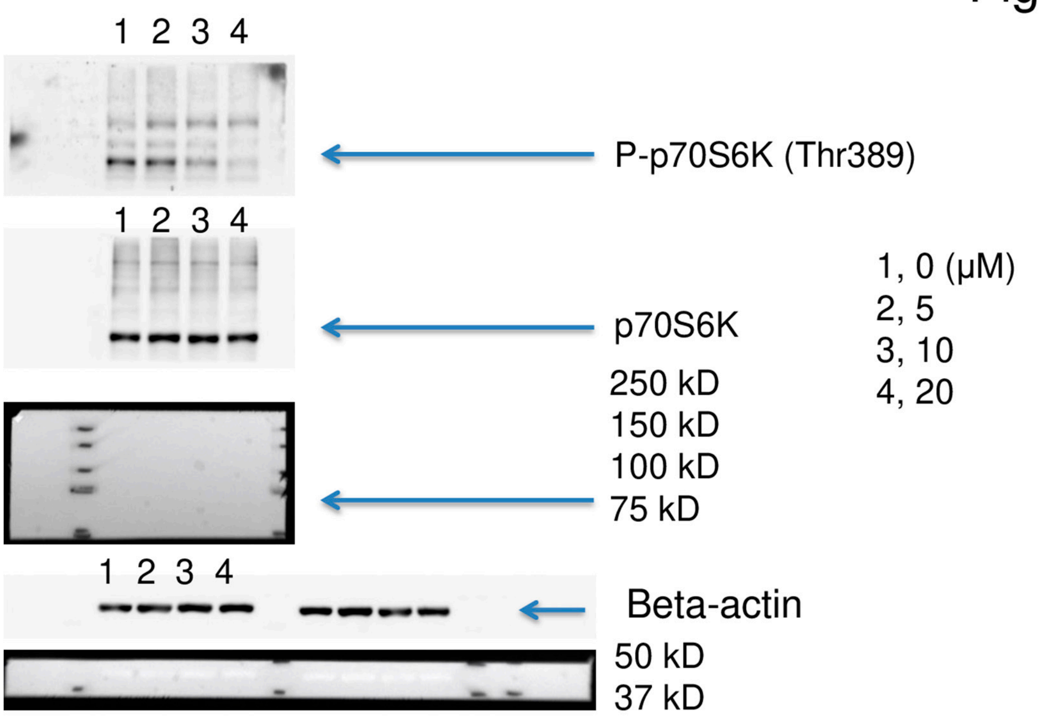

1 2 3 4
P-p70S6K (Thr389)
1 2 3 4
p70S6K
1, 0 (μM)
2, 5
3, 10
4, 20
250 kD
150 kD
100 kD
75 kD
1 2 3 4
Beta-actin
50 kD
37 kD

Fig. 2F

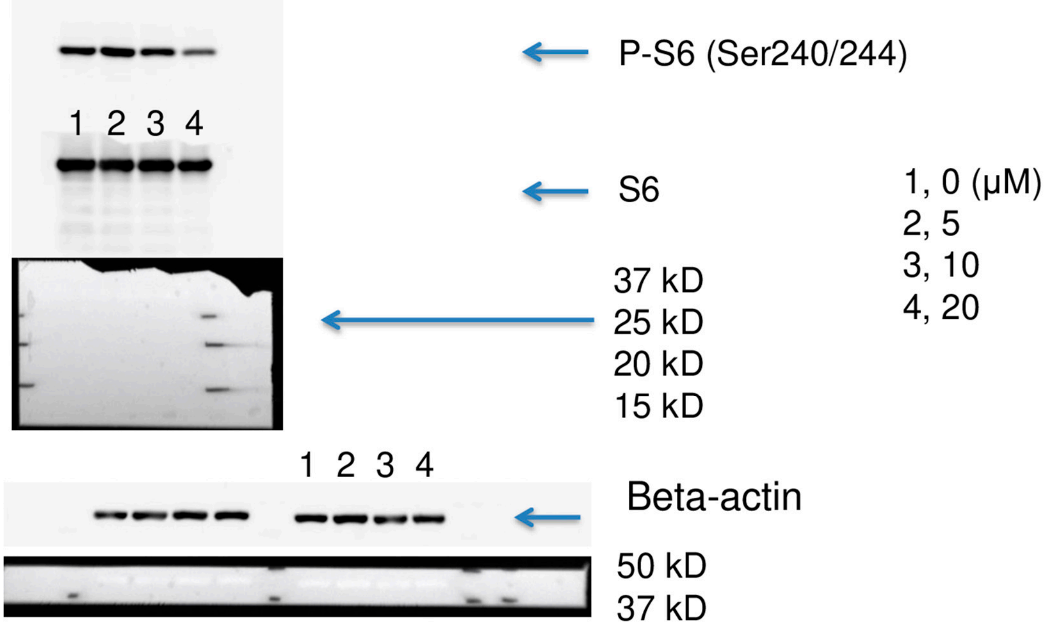

1 2 3 4
P-S6 (Ser240/244)
1 2 3 4
S6
1, 0 (μM)
2, 5
3, 10
4, 20
37 kD
25 kD
20 kD
15 kD
1 2 3 4
Beta-actin
50 kD
37 kD

Fig. 2G

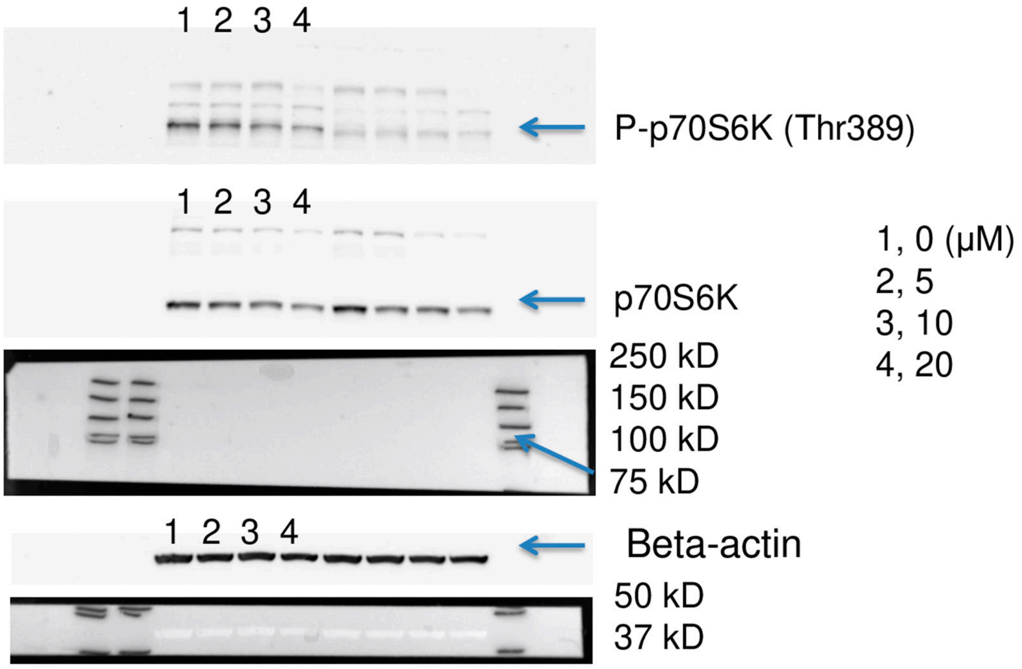
1 2 3 4
P-p70S6K (Thr389)
1 2 3 4
p70S6K
1, 0 (μM)
2, 5
3, 10
4, 20
250 kD
150 kD
100 kD
75 kD
1 2 3 4
Beta-actin
50 kD
37 kD

Fig. 2H

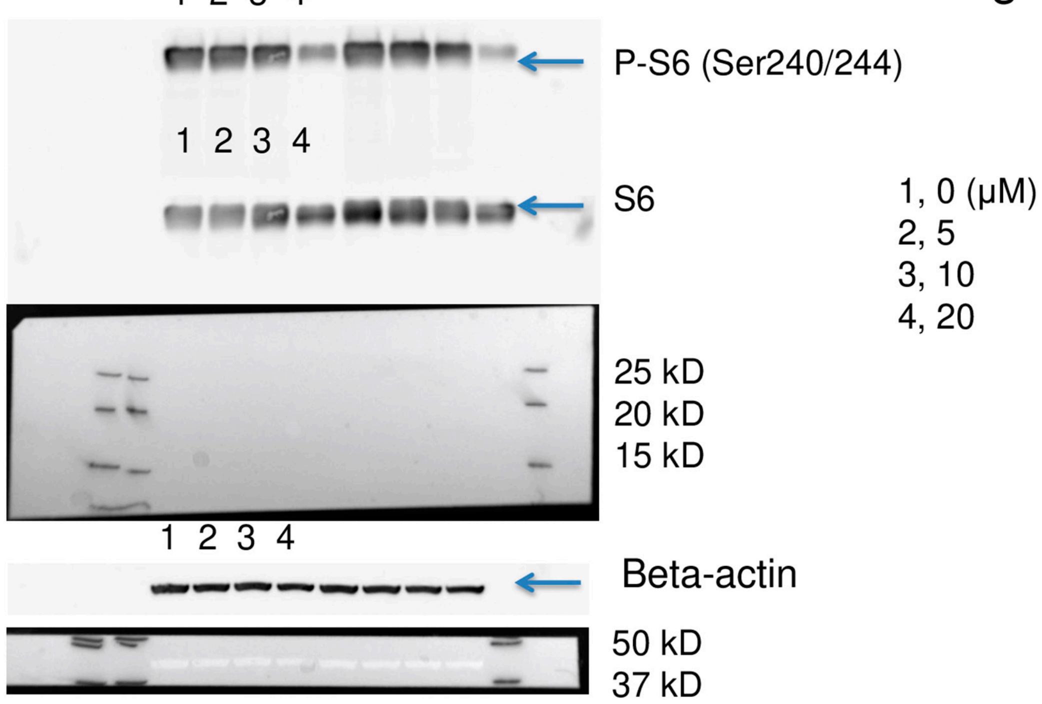
1 2 3 4
P-S6 (Ser240/244)
1 2 3 4
S6
1, 0 (μM)
2, 5
3, 10
4, 20
25 kD
20 kD
15 kD
1 2 3 4
Beta-actin
50 kD
37 kD

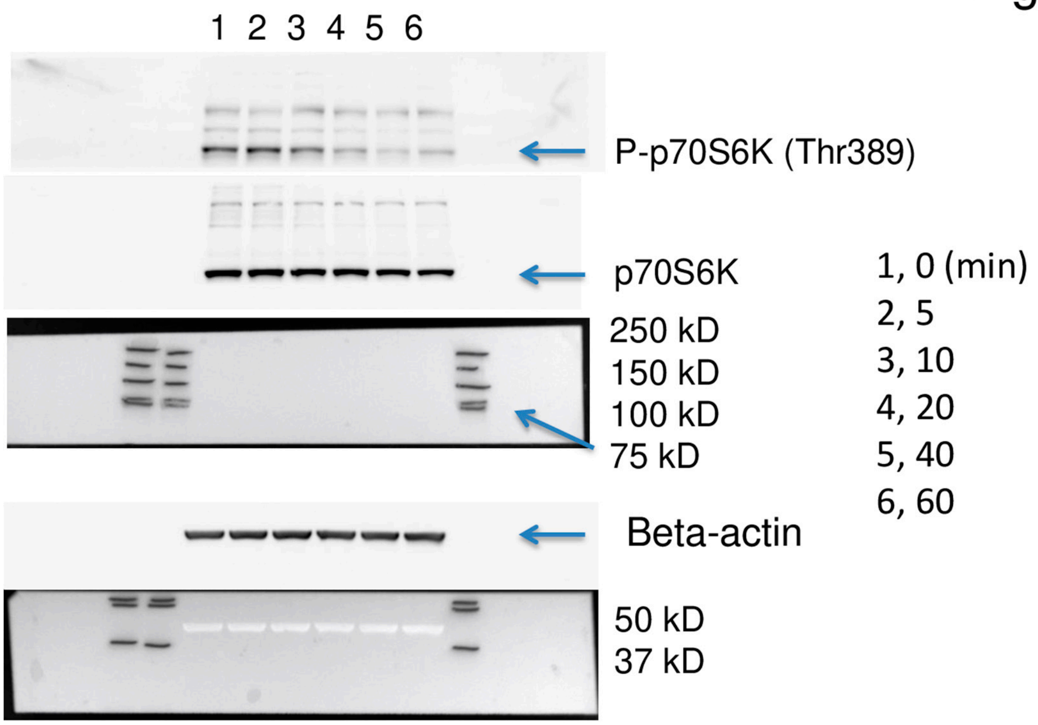

1 2 3 4 5 6
P-p70S6K (Thr389)
p70S6K
250 kD
150 kD
100 kD
75 kD
Beta-actin
50 kD
37 kD
1, 0 (min)
2, 5
3, 10
4, 20
5, 40
6, 60

Fig. 3A

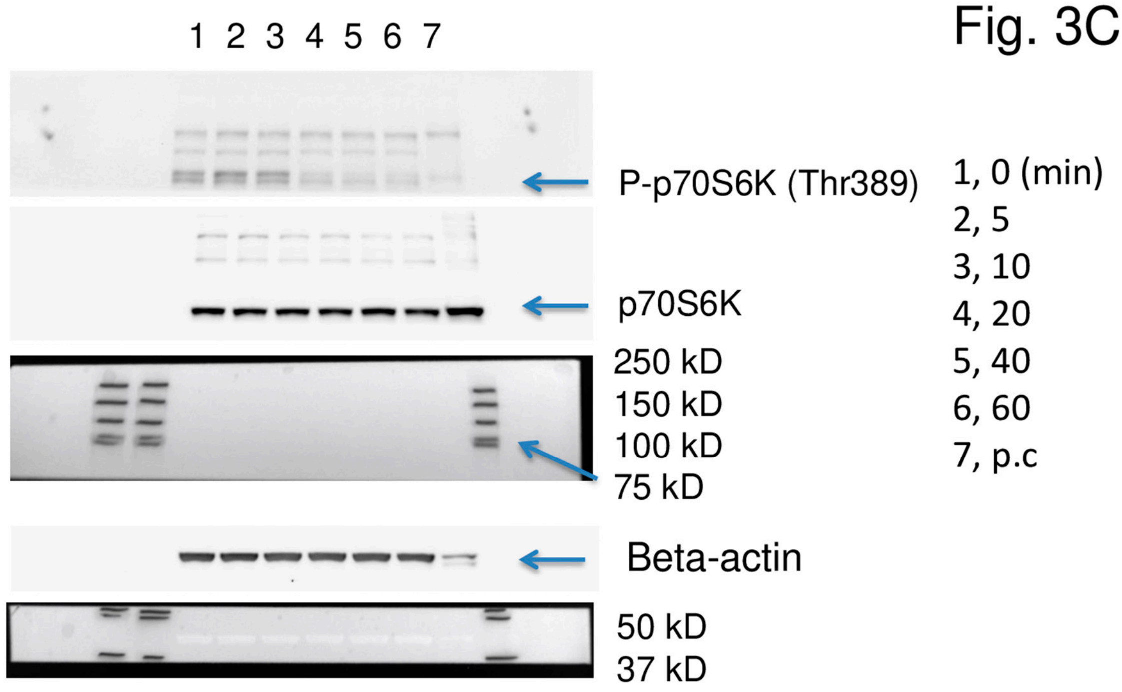

1 2 3 4 5 6 7
P-p70S6K (Thr389)
p70S6K
250 kD
150 kD
100 kD
75 kD
Beta-actin
50 kD
37 kD
1, 0 (min)
2, 5
3, 10
4, 20
5, 40
6, 60
7, p.c

Fig. 3C

Fig. 3E

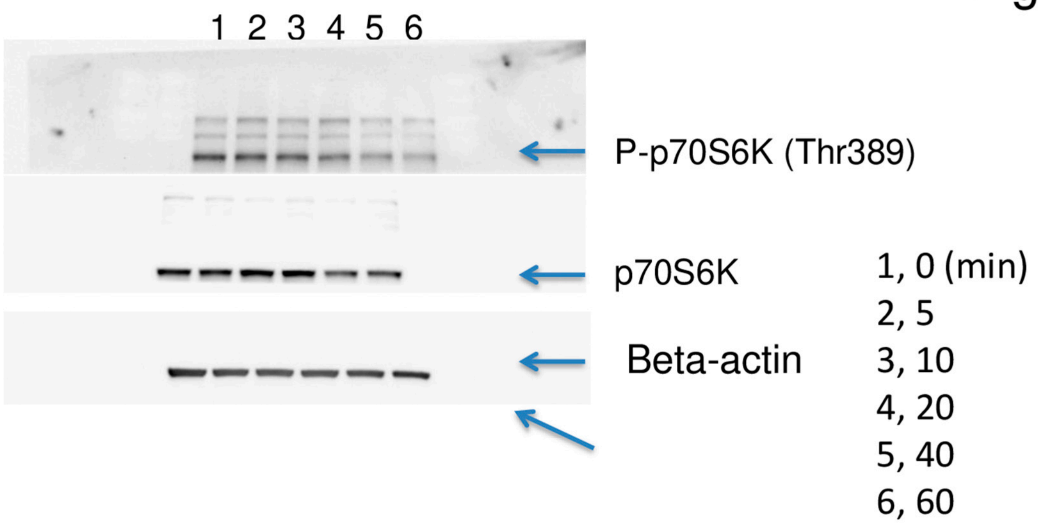
1 2 3 4 5 6
P-p70S6K (Thr389)
p70S6K
Beta-actin
1, 0 (min)
2, 5
3, 10
4, 20
5, 40
6, 60

Fig. 3G

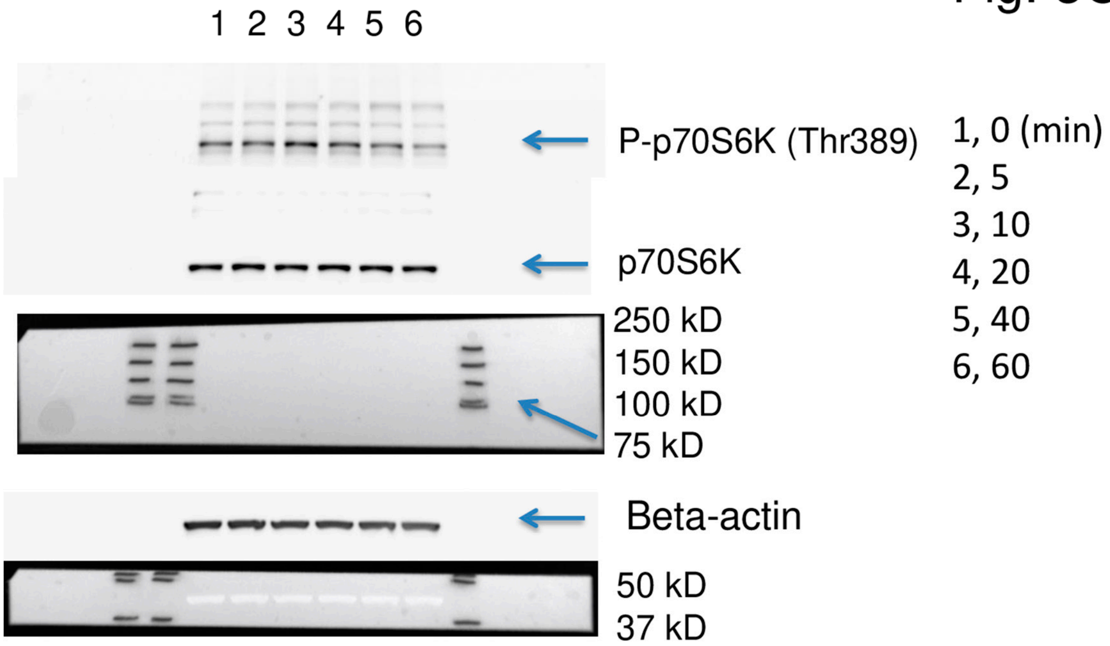
1 2 3 4 5 6
P-p70S6K (Thr389)
p70S6K
250 kD
150 kD
100 kD
75 kD
Beta-actin
50 kD
37 kD
1, 0 (min)
2, 5
3, 10
4, 20
5, 40
6, 60

Fig. 4A

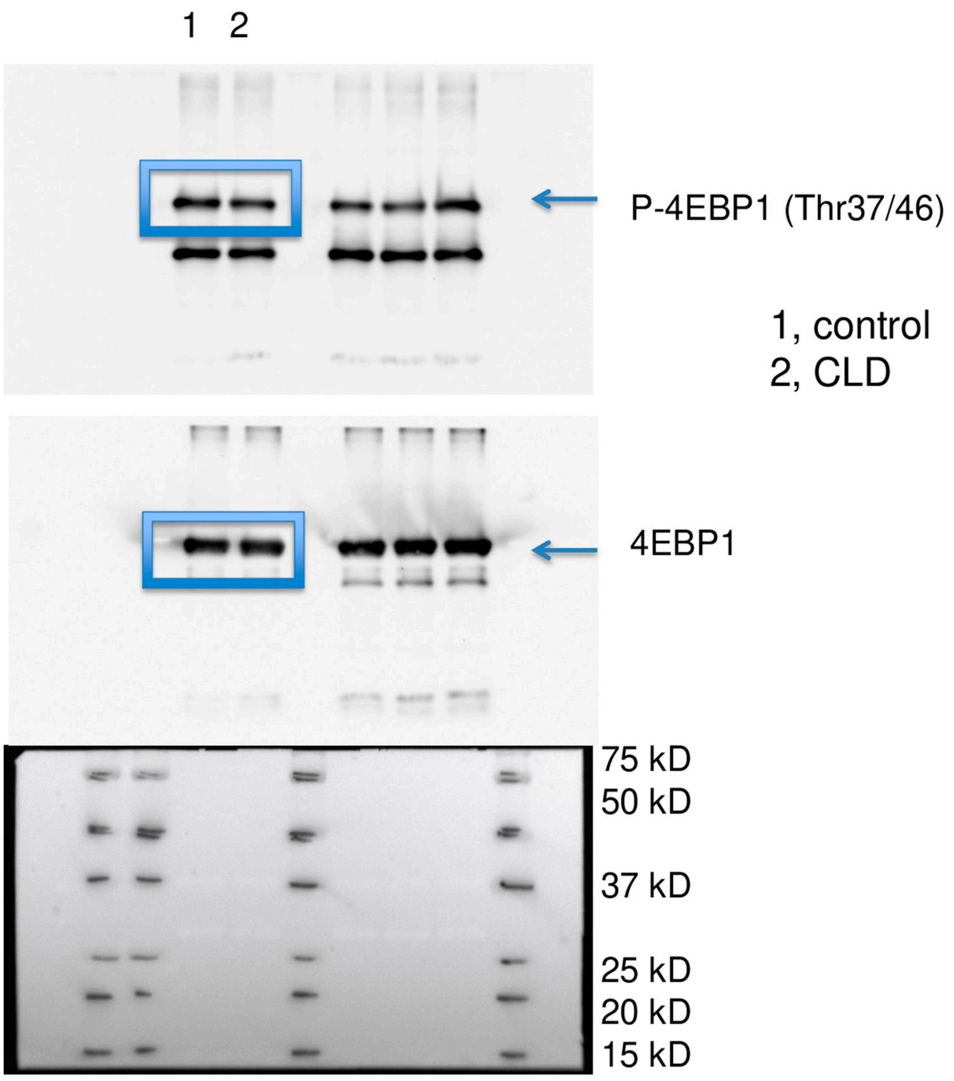
1
2
P-4EBP1 (Thr37/46)
1, control
2, CLD
4EBP1
75 kD
50 kD
37 kD
25 kD
20 kD
15 kD

Fig. 4E

Cyclin D1

1 2 3

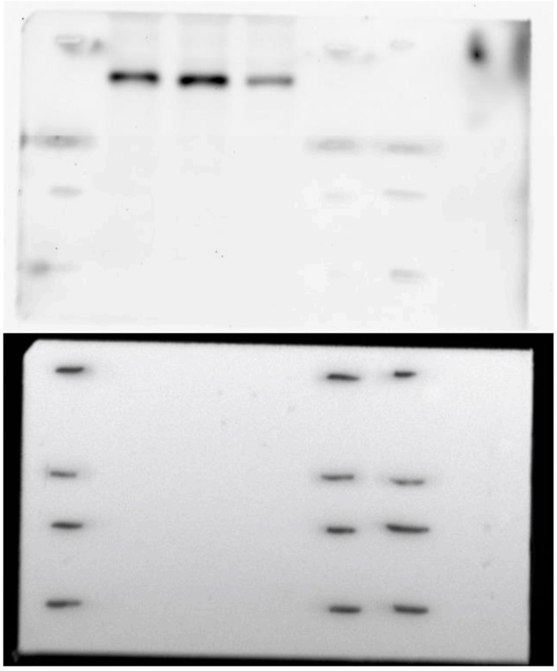

37 kD
25 kD
20 kD
15 kD

1, no treatment
2, vehicle (24 h)
3, CLD (24 h)

Beta-actin

1 2 3

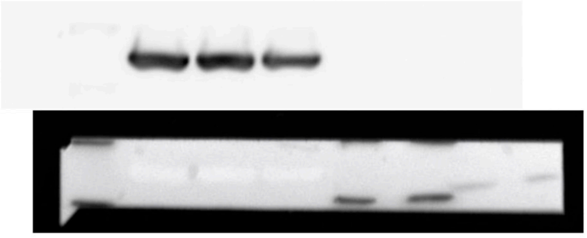

50 kD
37 kD

Fig. 5E

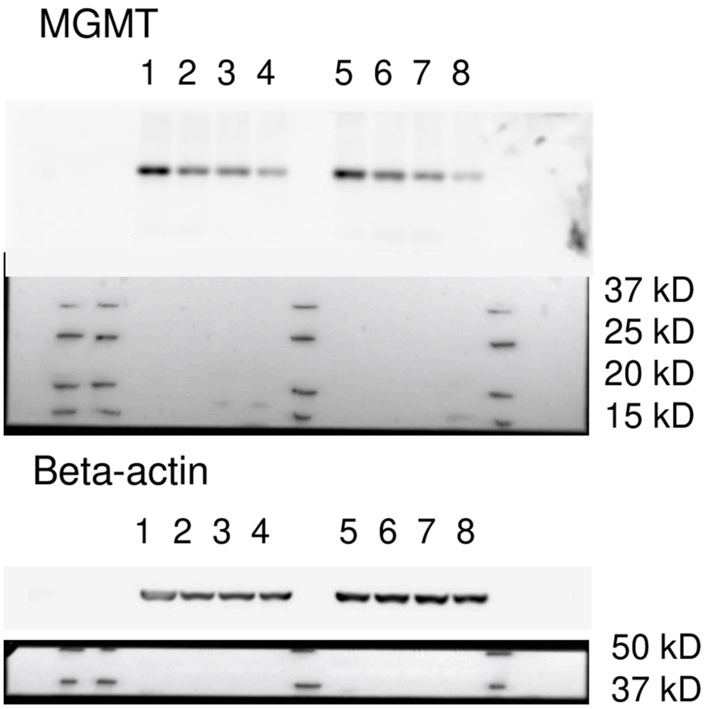

1, con
2, CLD
3, TMZ
4, CLD + TMZ
5, con
6, CLD
7, TMZ
8, CLD + TMZ

Fig. 5F

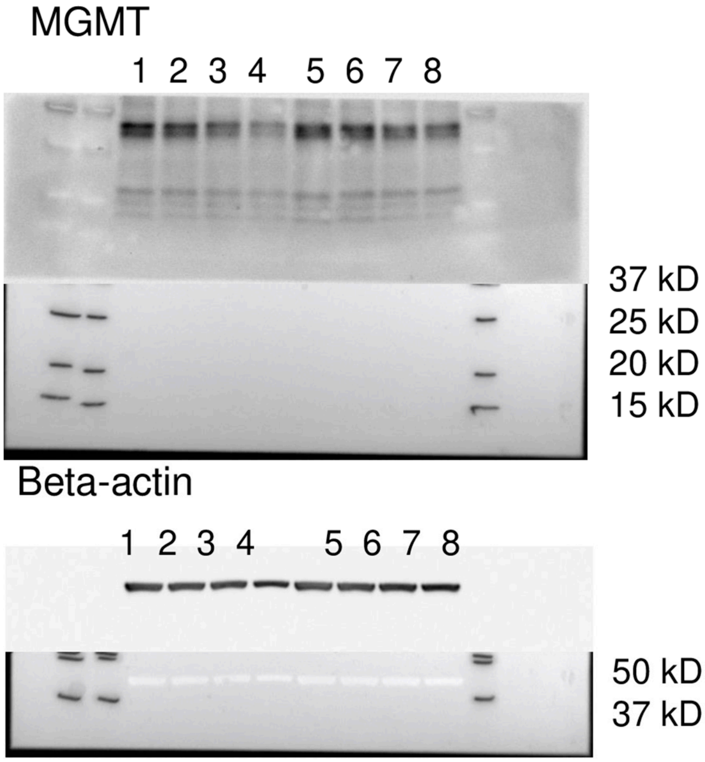

Fig. 6

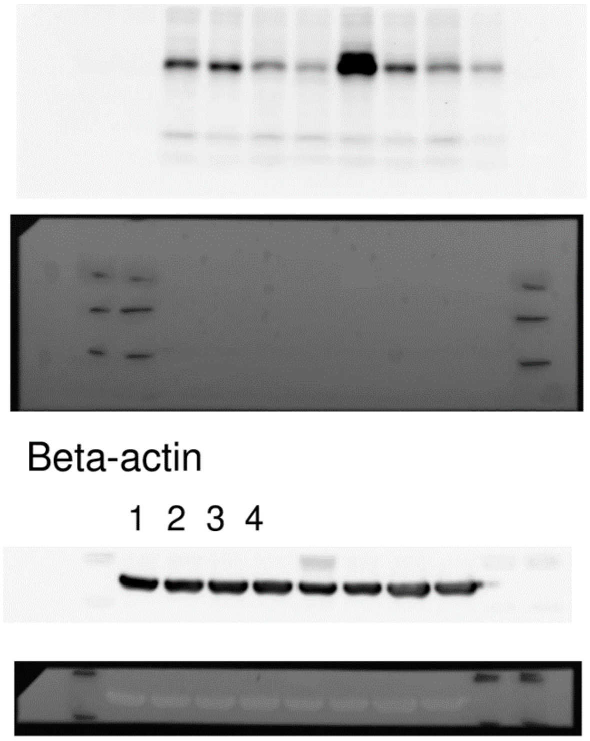
MGMT
1 2 3 4
1, con
2, CLD
3, TMZ
4, CLD + TMZ
25 kD
20 kD
15 kD
Beta-actin
1 2 3 4
50 kD
37 kD
